# Study protocol of a multicenter randomized controlled trial of mindfulness-based intervention versus relaxation to reduce emotional exhaustion in medical students in France: the “Must prevent” study

**DOI:** 10.1186/s12888-020-02529-9

**Published:** 2020-03-11

**Authors:** Carolina Baeza-Velasco, Catherine Genty, Isabelle Jaussent, Myriam Benramdane, Philippe Courtet, Emilie Olié

**Affiliations:** 1 Department of Emergency Psychiatry and Acute Care, CHU Montpellier, France. 191 Av du Doyen Gaston Giraud, 34090 Montpellier, France; 2 University of Paris, Laboratory of Psychopathology and Health Processes, F-92100 Boulogne-Billancourt, France; 3 PSNREC, University of Montpellier, INSERM, Montpellier, France

**Keywords:** Medical students, Mindfulness, Relaxation, Emotional exhaustion, Psychopathology, Empathy

## Abstract

**Background:**

Medical students are exposed to an emotionally exhausting training/work environment and to stressful academic demands. Consequently, psychopathologies, burnout and suicidal ideation are frequent in this population. These factors can also affect their empathy and quality of care. Therefore, the development and implementation of programs to promote resilience to stress specifically in medical students and the evaluation of their efficiency are a priority. Here, we describe the protocol of the first French study to assess the long-term effectiveness and acceptability of a mindfulness-based intervention (MBI) compared with relaxation training (RT) to reduce emotional exhaustion in medical students.

**Methods:**

This multicenter randomized controlled trial (“Must prevent”) plans to enroll 612 students in the fourth and fifth year of medical studies from nine French universities. After inclusion, they will be assigned randomly to the MBI or RT group. Both interventions are structured around an 8-week program that includes one group class per week and daily at-home exercises. The primary endpoint is the emotional exhaustion score assessed with the Maslach Burnout Inventory at month 12 of the follow-up. Secondary endpoints include anxiety-depressive symptomatology, suicidality, psychoactive substance use, depersonalization, psychological and physical pain, empathy, emotional regulation, self-compassion, mindfulness, quality of life, and program acceptability. Evaluations will be done before and immediately after the 8-week intervention, and at month 6 and 12 of the post-intervention follow-up.

**Discussion:**

If the proposed interventions are well accepted and useful to decrease negative emotions and/or increase wellbeing among medical students, they should be disseminated among this population and even included as part of the training on emotional skills needed for the routine medical practice.

**Trial registration:**

This trial is registered under the number NCT04026594 (July 18, 2019).

## Background

Medical students are exposed to challenging academic demands and to an emotionally exhausting work environment. Consequently, their levels of psychological distress, particularly depression, pathological anxiety, burnout, and suicidal behavior, are higher than in the general population [1–3]. Psychological suffering is rarely verbalized by medical students because this is often associated with weakness, and perceived as a barrier to medical practice [4]. Thus, psychopathology and burnout may persist after the end of the medical studies [5], leading young physicians to begin their careers in suboptimal conditions which may affect service delivery and quality of care [6]. A recent survey on the health of medical students and young doctors in France (*N* = 21,768) illustrate well the above. Results revealed that 68.2% of participants reported pathologic anxiety, 27.7% high level of depressive symptomatology, and 23.7% suicidal ideation [7].

Moreover, the stress of the academic and psychological demands can impair affective abilities, such as compassion and empathy that are important factors for optimal care delivery and successful social interactions in medical settings [8, 9]. There is evidence that empathy tends to decrease during the medical training period [10], compromising the communication with patients and ultimately the physician’s competence. In this context, the development and implementation of prevention programs to promote stress resilience and to develop emotional competences among medical students is a priority.

Mindfulness-Based Interventions (MBI) favor an intentional and non-judgmental awareness of the present moment [11]. Several studies have shown that MBIs have a positive effect on mental health outcomes and on the development of stress management skills and emotional competences [12]. The systematic reviews by O’Driscoll et al. [13] and by McConville et al. [14] explored MBI effect on students’ health and social care and reported positive changes on mood, anxiety, mindfulness levels, well-being, self-efficacy, empathy, self-compassion and coping abilities. Moreover, MBIs are well accepted and even popular among university students, probably because these approaches are perceived as emotional skill training strategies rather than programs addressed to improve mental health outcomes [15].

Relaxation training (RT) is another effective approach to manage stress in different populations [16–18]. Studies on student populations have shown that RT contributes to decrease distress, academic stress, test anxiety, and to increase positive mood [19–21].

Few studies (and none in France) compared MBI efficacy in medical students and in an active control group [13], and only one compared MBI and RT in this population. Jain et al. [20] evaluated the effectiveness of an adaptation of the Mindfulness-Based Stress Reduction (MBSR) program developed by Kabat-Zinn [22] compared with somatic relaxation in students pursuing different medical degrees (*N* = 83). They found that both interventions reduced distress and increased positive mood; however, the MBSR program was more effective in increasing positive states of mind. These preliminary results should be confirmed in larger samples. New studies should include also a long-term follow-up to explore the maintenance of such benefits, which is currently unknown [13].

Here, we describe the protocol of the first multicenter, randomized, controlled trial with one year of follow-up to compare MBI and RT effectiveness in reducing emotional exhaustion and in promoting mental health and emotional skills in medical students in France.

### Aims

The main objective of the “Must Prevent” trial is to evaluate and compare the effectiveness of MBI and RT in reducing emotional exhaustion in fourth- and fifth-year medical students at month 12 of the follow-up after the end of the intervention.

The secondary objectives are:

- to compare psychopathological parameters (i.e. anxiety-depressive symptomatology, perceived stress, psychoactive substances consumption, psychological pain, depersonalization, and suicidality), emotional skills (i.e. mindfulness levels, empathy, self-compassion, emotional regulation), and quality of life before and immediately after the intervention, and then at month 6 and 12 of the post-intervention follow-up.
- to investigate the acceptability and satisfaction of these interventions.

We hypothesized that both interventions will be useful to decrease emotional exhaustion and psychopathological parameters in medical students. However, we think that MBI will be more effective than RT for developing emotional skills, such as empathy, self-compassion and mindfulness.

## Methods

### Design and participants

This is a multicenter, single-blind, randomized, and controlled study. The main inclusion criterion is to be a fourth- or fifth-year medical student. Exclusion criteria include: current depressive episode and/or panic disorder according to the DSM-5 diagnostic classification [23], and refusal to participate.

**Fig. 1:**
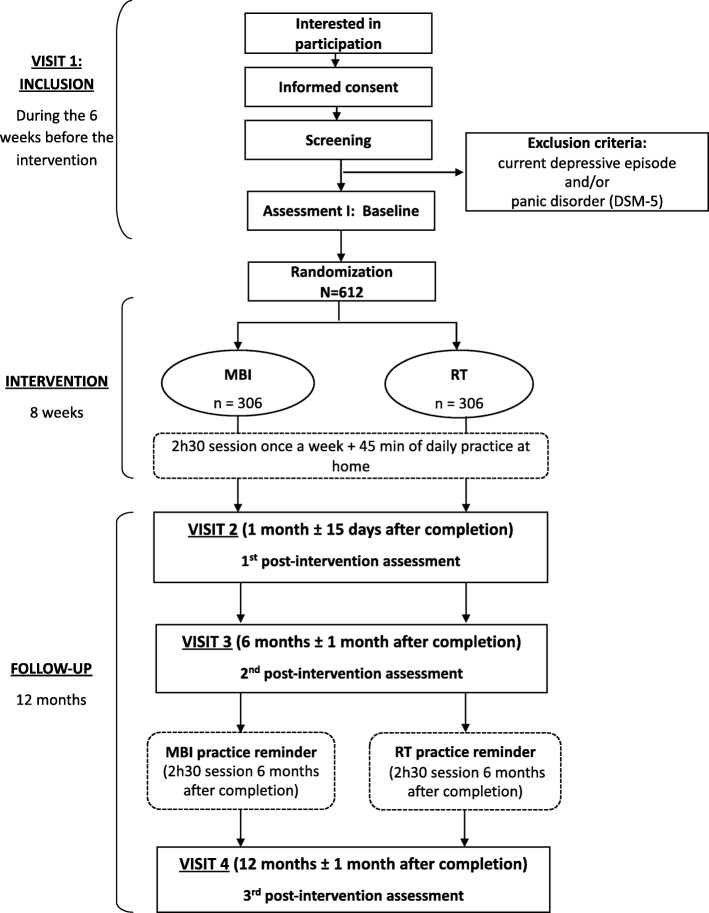
Study Design

The study protocol was registered in the Clinical Trials Registry (ClinicalTrials.gov; number NCT04026594, July 18, 2019) and was authorized by the French Health Ministry (ANSM, September 13, 2019), and approved by the French Est I Ethics Committee for the Protection of Patients (June 20, 2019). Figure 1 summarizes the study design.

### Sample size

The sample size calculation was based on the results of the work by Verweij et al. [24]. In this randomized controlled trial, the mean baseline score of the emotional exhaustion subscale of the Maslach Burnout Inventory (MBIn) [25]. of 148 residents working in one of the medical, surgical or primary care disciplines was 15.6 (SD = 7.5). Assuming that the baseline score distribution will remain unchanged at the end of the trial in the MBI group, 274 students per group are required to show a significant mean between-group difference of 1.8 points for the emotional exhaustion subscale score at the end of the trial (15.6 for the MBI group and 17.4 for the RI group) with a statistic power of 80% and a 2-sided α level of 0.05. The percentage of participants lost to follow-up is estimated at 10%. Therefore, in total 612 students need to be recruited in the nine universities participating in the trial.

### Procedure

Recruitment will take place in nine French medical universities (Montpellier, Paris, Marseille, Nîmes, Strasbourg, Clermont-Ferrand, St Etienne, Angers and Tours) where the study will be advertised and students will receive general information. Students interested in participating will be included after verification of their eligibility and after signing an informed consent form obtained via the investigators. Participants will be randomized to the MBI or the RT group by an independent researcher, not otherwise involved in the study. Randomization will be stratified by study center and degree of emotional exhaustion (MBIn emotional exhaustion subscale score < 17, 18–29, and ≥ 30 [25, 26]). Randomization will be centralized, accessible online and programmed using the Capture Software System (EnnovClinical randomization module) according to a minimization algorithm with a 1:1 ratio.

Participants not included in the study because of a current depressive episode or panic disorder will be referred to the psychological unit of their university.

Participants will be evaluated at four time points (Fig. 1): before (i.e. within 6 weeks) the intervention (visit 1), within the month after the intervention end (visit 2), and at month 6 (visit 3) and month 12 (±1 month; visit 4) of the post-intervention follow-up. Raters will be blind to the allocation (simple-blind). Research assistants and MBI/RT instructors will emphasize the importance of blinding to participants and will repeatedly remind them not to reveal their intervention type. In case of unblinding, another assessor will repeat the entire measurement.

A monetary compensation (40 euros) will be given to participants after each follow-up visit (visits 2 to 4) for the time devoted to the study.

### MBI and RI programs

The MBI is an adaptation of the MBSR program developed by Kabat-Zinn [22]. The intervention will last 8 weeks, with one group session of 150 min per week. It will be delivered according to the fundamental concepts and specific techniques provided in the MBSR manual (e.g. body scan, hatha yoga, sitting, walking, and loving kindness meditation). It will also include up to 45 min of daily exercises at home, but not the full-day silent retreat programmed at week 6 of the course in the original protocol. This retreat will not be done for reasons of feasibility, and to have a similar number of training hours in both groups (MBI and RT). To adapt the program to medical students, discussions on mindfulness and related topics will be contextualized to the specific academic/medical demands.

The 8-week RT program includes one group session of 150 min per week and 45 min of daily at-home exercises. After an introduction about relaxation and its usefulness, participants will learn relaxation techniques classified in three types: relaxing breathing techniques (e.g. abdominal breathing), muscle relaxation (e.g. Jacobson’s progressive relaxation technique), and cognitive relaxation (e.g. guided imageries).

In both interventions, participants are encouraged to perform training exercises at home every day and to fill in worksheets to keep a record of their training and promote self-observation. A recall MBI and RT group session will be proposed at month 6 after the intervention end to strengthen their motivation and to avoid forgetting the techniques.

MBI and RT group sessions will be carried out by accredited (in the case of MBSR) and experienced instructors (two instructors per center).

Although no risks are expected for both interventions, participants will be encouraged on enrolment to look for signs of their health deteriorating, related or no to MBI/RT interventions. If any physical or mental symptom emerges, participants will be referred to relevant health services and/or the research team will contact the preventive medicine of the concerned faculty.

### Assessments

At visit 1, the Mini International Neuropsychiatric Interview (MINI 7 [27]) will be administered to assess mental disorders according to the DSM-5 criteria and to identify participants with current major depression or panic disorder (exclusion criteria). In addition, depression intensity will be assessed with the Montgomery Asberg Depression Scale (MADRS [28, 29]), and suicidality using the Columbia–Suicide Severity Rating Scale, (C-SSRS [30]). Data on the participants’ sociodemographic characteristics (i.e. age, sex, marital status, educational level), and access to psychiatric care (i.e. past psychiatric hospitalization, previous visit to a psychiatrist, psychologist or general practitioner for psychological reasons, previous psychotherapy, and current medications) will also be collected.

In addition to this hetero-evaluation, participants will be asked to complete a set of self-questionnaires to assess the primary and secondary endpoints:

#### Primary endpoint measure

- MBIn [25, 26]) emotional exhaustion subscale, which represents the primary endpoint.

#### Secondary endpoint measures

- MBIn depersonalization and professional achievement subscales,
- Hospital Anxiety and Depression Scale (HADS [31, 32]),
- *Perceived Stress Scale* (PSS [33, 34]),
- Difficulties in Emotional Regulation Scale (DERS [35, 36]),
- Fagerstrom Test for Cigarette Dependence (FTCD [37, 38]),
- Alcohol Use Disorders Identification Test (AUDIT [39, 40]),
- Drug Abuse Screening Test (DAST [41, 42]),
- Cannabis Abuse Screening Test (CAST [43]),
- Jefferson Scale of Empathy, student version (JSEs [44, 45]),
- Five Facet Mindfulness Questionnaire (FFMQ [46, 47]),
- Self-Compassion Scale (SCS [48, 49]),
- World Health Organization Quality of Life (WHOQOL-BREF [50, 51]),
- Numerical scales (from 0 to 10) for current and worst (during the last 15 days) psychological and physical pain, and for suicidal ideation.

The acceptability of the two interventions will be assessed through the number of missed sessions, and satisfaction using a Likert scale from 0 (not useful at all) to 10 (extremely useful). Table 1 summarizes the assessments performed at each visit.

**Table 1:** Assessments at the different time points

Instrument	Target	Visit 1 Inclusion	Visit 2 Within the 1st month post-intervention	Visit 3 6 months post-intervention	Visit 4 12 months post-intervention
MINI 7	Lifetime psychiatric disorders	X			
MADRS	Depression	X	X	X	X
C-SSRS	Suicidality	X	X	X	X
MBIn	Emotional exhaustion, Depersonalization & Professional achievement	X	X	X	X
Fagerstrom	Tobacco use	X			X
AUDIT	Alcohol use	X			X
DAST	Illicit substances use	X			X
CAST	Cannabis use	X			X
HADS	Anxiety-depressive symptomatology	X	X	X	X
PSS	Perceived stress	X	X	X	X
Numerical scales	Psychological pain Physical pain Suicidal ideation	X	X	X	X
Numerical scale	Intervention satisfaction		X		
JSEs	Empathy	X	X	X	X
DERS	Emotional regulation	X	X	X	X
IRI	Interpersonal reactivity	X			X
WHOQoL-BREF	Quality of life	X			X
FFMQ	Mindfulness skills	X	X		X
SCS	Self-compassion	X	X		X

*MINI 7* International Neuropsychiatric Interview, *MADRS* Montgomery Asberg Depression Scale, *C-SSRS* Columbia Suicide Severity Rating scale, *MBIn* Maslach Burnout Inventory, *AUDIT* Alcohol Use Disorders Identification Test, *DAST* Drug Abuse Screening Test, *CAST* Cannabis Abuse Screening Test, *HADS* Hospital Anxiety and Depression Scale, *PSS* Perceived Stress Scale, *JSE* Jefferson scale of empathy student version, *DERS* Difficulties in Emotional Regulation Scale, *IRI* Interpersonal reactivity index, *WHOOQL-BREF* World Health Organization Quality of Life, *FFMQ* Five Facet Mindfulness Questionnaire, *SCS* Self-compassion scale

### Statistical analyses

Demographic data and baseline characteristics will be presented for the whole population and for each intervention group. Categorical variables will be described as numbers and percentages, and quantitative variables as mean and standard deviation or median and range, according to their distribution (normal or not).

The MBIn emotional exhaustion subscale score (primary endpoint) will be compared in the two groups at the end of the trial. An analysis of covariance will be performed including the baseline emotional exhaustion subscale score and baseline covariates that are found to be significantly different in between-group comparisons. If the conditions for carrying out a covariance analysis are not met, the continuous variable will be dichotomized and logistic regression models will be implemented.

The same methodology will be used to analyze the secondary endpoints at visit 4.

Mixed models will be used to study variations (intra- and between-group) of each variable during the follow-up. All statistical analyses will be performed using SAS® (SAS Institute, Cary, NC, USA) and the significance level will be set at *p* < 0.05.

#### Dissemination

News with regards to the implementation of the Must prevent study will be communicated to relevant parties by a quarterly newsletter. Trial results will be communicated by publishing scientific articles.

## Trial status

The recruitment for the Must Prevent study started in October 2019 and is still ongoing at the time of this manuscript submission.

## Discussion

Despite international and national recent data alerting about the psychological suffering experienced by medical students, in France no study so far has evaluated the effectiveness of an intervention for reducing negative emotions in this population. The Must Prevent study seeks to fill this gap by proposing the implementation and evaluation of two well-known interventions (MBI and RT) to reduce stress in a large sample of fourth- and fifth-year medical students from different Universities. Although some isolated experiences on MBI in some French universities have been reported, no collaborative study including medical schools from different cities exists. Furthermore, several research on MBI efficacy in this population has been performed with students in more advanced courses or already graduated (e.g. [52, 53]). Yet, it is important to explore the benefits of starting interventions earlier to improve stress resilience already during the first years of medical education. Additionally, most previous studies did not have a long-term follow-up. Conversely, our study includes 1 year of follow-up.

To integrate the MBI or RT program in their routine and to obtain benefits, students will have to be actively engaged and to regularly practice the intervention at home. As *medical* studies are very *time-consuming,* it is relevant to explore the participants’ capacity to integrate this activity in their routine. According to Ishak et al. [5], wellness activities can be perceived by some students as another obligation to fulfill. Thus, the acceptability and adherence to these interventions will also be evaluated. Previous research has highlighted the popularity of these approaches among university students [15]. However, this finding must be validated in the specific context of medical training. If the benefits and acceptability are confirmed, there will be cumulative scientific arguments for incorporating MBI and/or RT in the medical curriculum. As Moir et al. [54] stated, “the advantages of the addition of a well-being curriculum, as skills to prevent and manage distress and depression are relevant in supporting the competencies required by medical practitioners”.

Moreover, the introduction in medical schools of mind-body approaches, such as mindfulness and relaxation, might be useful to help relativize the outdated Cartesian dualism still popular in the medical culture. This will also contribute to raise awareness among students that in addition to knowledge and technical competences, emotional and relational skills are also necessary for an optimal medical practice.

One important limitation of this study will be the potential self-selection bias. Indeed, students who will participate in the trial are those motivated and/or particularly open to psychological approaches. Nevertheless, this project will bring new data on the usefulness of MBI and RT programs to develop psychological resources in medical students for better coping with stressful academics demands, and with an emotionally exhausting work environment. This line of research is relevant because it addresses the mental health problem of a specific population who benefits from few or no targeted intervention to cope with and reduce the high levels of stress.

## Data Availability

Research data will be stored, managed and monitored at the CHU of Montpellier. Data use or transmission to a third party may be done with its prior agreement.

## Abbreviations

AUDIT: Alcohol Use Disorders Identification Test
CAST: Cannabis Abuse Screening Test
C-SSRS: Columbia Suicide Severity Rating scale
DAST: Drug Abuse Screening Test
DERS: Difficulties in Emotional Regulation Scale
DSM-5: Diagnostic and Statistical Manual of Mental Disorders, Fifth Edition
FFMQ: Five Facet Mindfulness Questionnaire
HADS: Hospital Anxiety and Depression Scale
IRI: Interpersonal Reactivity Index
JSEs: Jefferson Scale of Empathy (student)
MADRS: Montgomery Asberg Depression Scale
MBI: Mindfulness Based Intervention
MBIn: Maslach Burnout Inventory
MBSR: Mindfulness-Based Stress Reduction
Mini: International Neuropsychiatric Interview
PSS: Perceived Stress Scale
RT: Relaxation Training
SCS: Self-Compassion Scale
WHOOQL-BREF: World Health Organization Quality of Life

## References

[1] Dyrbye LN, Thomas MR, Shanafelt TD (2006). Systematic review of depression, anxiety, and other indicators of psychological distress among U.S. and Canadian medical students. Acad Med.

[2] Dyrbye L, Shanafelt T (2016). A narrative review on burnout experienced by medical students and residents. Med Educ.

[3] Rotenstein L, Ramos M, Torre M (2016). Prevalence of depression, depressive symptoms and suicidal ideation among medical students. A systematic review and meta-analysis. JAMA.

[4] Kothari V, George N, Hamid O (2018). Provision of mental health support for medical students. Adv Med Educ Pract.

[5] Ishak W, Nikravesh R, Lederer S, Perry R, Ogunyemi D, Bernstein C (2013). Burnout in medical students: a systematic review. Clin Teach.

[6] Rabin S, Fieldman D, Kaplan Z (1999). Stress and intervention strategies in mental health professionals. Br J Med Psychol.

[7] Syndicats ANEMF, ISNI, ISNAR-IMG, ISNCCA. Enquête Santé Mentale Jeunes Médecins 2017. Available at www.isnar-img.com/wp-content/uploads/ESMJM_dossier_de_presse.pdf..

[8] Sinclair S, McClement S, Raffin-Bouchal S, Hack TF, Hagen NA, McConnell S, Chochinov HM (2016). Compassion in health care: an empirical model. J Pain Symptom Manag.

[9] Ogle J, Bushnell JA, Caputi P (2013). Empathy is related to clinical competence in medical care. Med Educ.

[10] Hojat M, Vergare MJ, Maxwell K (2009). The devil is in the third year: a longitudinal study of erosion of empathy in medical school. Acad Med.

[11] Kabat-Zinn J (1990). Full catastrophe living: using the wisdom of your body and mind to face stress, pain and illness.

[12] Chiesa A, Serretti A (2009). Mindfulness-based stress reduction for stress management in healthy people: a review and a meta-analysis. J Altern Complement Med.

[13] O'Driscoll M, Byrne S, Mc Gillicuddy A, Lambert S, Sahm LJ (2017). The effects of mindfulness-based interventions for health and social care undergraduate students - a systematic review of the literature. Psychol Health Med.

[14] McConville J, McAleer R, Hahne A (2017). Mindfulness training for health profession students- the effect of mindfulness training on psychological well-being, learning and clinical performance of health professional students: a systematic review of randomized and non-randomized controlled trials. Explore.

[15] Galante J, Dufour G, Vainre M, Wagner AP, Stochl J, Benton A, Lathia N, Howarth E, Jones PB (2018). A mindfulness-based intervention to increase resilience to stress in university students (the mindful student study): a pragmatic randomized controlled trial. Lancet Public Health.

[16] Manzoni GM, Pagnini F, Castelnuovo G, Molinari E (2008). Relation training for anxiety: a ten years systematic review with meta-analysis. BMC Psychiatry.

[17] Volpato E, Banfi P, Rogers SM, Pagnini F. Relaxation techniques for people with chronic obstructive pulmonary disease: a systematic review and a meta-analysis. Evi Based Complement Alternat Med. 2015(8):628365. 10.1155/2015/628365. 10.1155/2015/628365 PMC4539049 26339268

[18] Yusufov M, Nicoloro-Santa Barbara J, Grey N, Moyer A, Lobel M (2019). Meta-analytic evaluation of stress reduction interventions for undergraduate and graduate students. Int J Stress Manag.

[19] Manansingh S, Tatum SL, Morote ES (2019). Effects of relaxation techniques on nursing students’s academic stress and test anxiety. J Nurs Educ.

[20] Jain S, Shapiro SL, Swanick S, Roesch SC, Mills PJ, Bell I, Schwartz G (2007). A randomized controlled trial of mindfulness meditation versus relaxation training; effects on distress, positive states of mind, rumination, and distraction. Ann Behav Med.

[21] Torabizadeh C, Bostani S, Yektatalab S (2016). Comparison between the effects of muscle relaxation and support groups on the anxiety of nursing students: a randomized controlled trial. Complement Ther Clin Pract.

[22] Kabat-Zinn J (1982). An outpatient program in behavioral medicine for chronic pain patients based on the practice of mindfulness meditation: theorical considerations and preliminary results. Gen Hosp Psychiatry.

[23] American Psychiatric Association (2013). Diagnostic and statistical manual of mental disorders.

[24] Verweij H, van Ravesteijn H, van Hooff MLM, Lagro-Janssen ALM, Speckens AEM (2018). Mindfulness-based stress reduction for residents: a randomized controlled trial. J Gen Intern Med.

[25] Maslach C, Jackson SE (1981). The measurement of experienced burnout. J Ocuppational Behav.

[26] Dion G, Tessier R (1994). Validation of the French translation of the Maslach burnout inventory (MBI). Can J Behav Sci.

[27] Lecrubier Y, Sheehan DV, Weiller E, Amorim P, Bonora I, Harnett Sheehan K, Janavs J, Dunbar GC (1997). The Mini International Neuropsychiatric Interview (MINI). A short diagnostic structured interview: reliability and validity according to the CIDI. Eur Psychiatry.

[28] Montgomery SA, Asberg M (1979). A new depression scale designed to be sensitive to change. Br J Psychiatry.

[29] Peyre F, Martinez R, Calache M, Verdoux H, Bourgeois M (1989). New validation of the Montgomery and Asberg depression scale (MADRS) on a sample of 147 hospitalized depressed patients. Ann Med Psychol.

[30] Posner K, Brown GK, Stanley B, Brent DA, Yershova KV, Oquendo MA, Currier GW, Melvin GA, Greenhill L, Shen S, Mann JJ (2011). The Columbia–suicide severity rating scale: initial validity and internal consistency findings from three multisite studies with adolescents and adults. Am J Psychiatry.

[31] Zigmond AS, Snaith RP (1983). The hospital anxiety and depression scale. Acta Psychiatr Scand.

[32] Bocéréan C, Dupret E (2014). A validation of the hospital anxiety and depression scale (HADS) in a large sample of French employees. BMC Psychiatry.

[33] Cohen S, Kamarck T, Mermelstein R (1983). A global measure of perceived stress. J Health Soc Behav.

[34] Bellinghausen L, Collange J, Botella M, Emery JL, Albert E (2009). Validation factorielle de l’échelle française de stress perçu en milieu professionnel. Santé Publique.

[35] Gratz KL, Roemer L (2004). Multidimensional assessment of emotion regulation and dysregulation: development, factor structure, and initial validation of the difficulties in emotion regulation scale. J Psychopathol Behav Assess.

[36] Dan-Glauser ES, Scherer KR (2013). The difficulties in emotion regulation scale (DERS): factor structure and consistency of a French translation. Swiss J Psychol.

[37] Fagerström K (2012). Determinants of tobacco use and renaming the FTND to the Fagerstrom test for cigarette dependence. Nicotine Tob Res.

[38] Etter JF, Duc TV, Perneger TV (1999). Validity of the Fagerström test for nicotine dependence and of the heaviness of smoking index among relatively light smokers. Addiciton.

[39] Saunders JB, Aasland OG, Babor TF, De la Fuente JR, Grant M (1993). Development of the alcohol use disorders identification test (AUDIT): WHO collaborative project on early detection of persons with harmful alcohol consumption-II. Addiction.

[40] Gache P, Michaud P, Landry U, Accietto C, Arfaoui S, Wenger O, Daeppen JB (2005). The alcohol use disorders indentification test (AUDIT) as a screening tool for excessive drinking in primary care: realiability and validity of a French version. Alcohol Clin Exp Res.

[41] Skinner HA (1982). The drug abuse screening test. Addict Behav.

[42] Centre de toxicomanie et de santé mentale (2011). Questionnaire sur la consommation de drogues (DAST-20).

[43] Legleye S, Karila L, Beck F, Reynaud M (2007). Validation of the CAST, a general population Cannabis abuse screening test. J Subst Use.

[44] Hojat M, Mangione S, Nasca T, Cohen M, Gonnella J, Erdmann J (2002). The Jefferson scale of empathy: development and preliminary psychometric data. Med Educ.

[45] Zenasni F, Boujut E, Bluffel de Vaure C, Catu-Pinault A, Tavani JL, Rigal L, Jaury P, Sultan S (2012). Development of a French-language version of the Jefferson scale of physician empathy and association with practice characteristics and burnout in a sample of general practitioners. Int J Pers Cent Med.

[46] Baer RA, Smith GT, Hopkins J, Krietemeyer J, Toney L (2006). Using self-report assessment methods to explore facets of mindfulness. Assessment.

[47] Heeren A, Doulliez C, Peschard V, Debrauwere L, Philippot P (2011). Cross-cultutal consistency of the five facet mindfulness questionnaire: adaptation and validation in a French sample. Eur Rev Appl Psychol.

[48] Neff KD (2003). Development and validation of a scale to measure self-compassion. Self Identity.

[49] Kotsou I, Leys C (2016). Self-compassion scale (SCS): psychometric properties of the French translation and its relations with psychological well-being, affect and depression. PLoS One.

[50] The WHOQOL Group (1998). Development of the World Health Organization WHOQOL-BREF quality of life assessment. Psychol Med.

[51] Baumann C, Erpelding ML, Régat S, Collin JF, Briançon S (2010). The WHOQOL-BREF questionnaire: French adult population norms for the physical health, psychological health and social relationship dimensions. Rev Epidemiol Sante Publique.

[52] Warnecke E, Quinn S, Ogden K, Towle N, Nelson MR (2011). A randomised controlled trial of the effects of mindfulness practice on medical student stress levels. Med Educ.

[53] Chung AS, Felber R, Han E, Mathew T, Rebillot K, Likourezos A (2018). A targeted mindfulness curriculum for medical students during their emergency medicine clerkship experience. West J Emerg Med.

[54] Moir F, Yielder J, Sanson J, Chen Y (2018). Depression in medical students: current insights. Adv Med Educ Pract.

